# Neurogenetic analysis of childhood disintegrative disorder

**DOI:** 10.1186/s13229-017-0133-0

**Published:** 2017-04-04

**Authors:** Abha R. Gupta, Alexander Westphal, Daniel Y. J. Yang, Catherine A. W. Sullivan, Jeffrey Eilbott, Samir Zaidi, Avery Voos, Brent C. Vander Wyk, Pam Ventola, Zainulabedin Waqar, Thomas V. Fernandez, A. Gulhan Ercan-Sencicek, Michael F. Walker, Murim Choi, Allison Schneider, Tammy Hedderly, Gillian Baird, Hannah Friedman, Cara Cordeaux, Alexandra Ristow, Frederick Shic, Fred R. Volkmar, Kevin A. Pelphrey

**Affiliations:** 1 Department of Pediatrics, Yale School of Medicine, New Haven, Connecticut USA; 2 Child Study Center, Yale School of Medicine, New Haven, Connecticut USA; 3 Department of Psychiatry, Yale School of Medicine, New Haven, Connecticut USA; 4 Department of Genetics, Yale School of Medicine, New Haven, Connecticut USA; 5 Evelina London Children’s Hospital, Guy’s and St. Thomas’ Trust, Kings Health Partners AHSC, London, UK

**Keywords:** Autism spectrum disorder (ASD), Childhood disintegrative disorder (CDD), Regression, Intellectual disability (ID), Genetics, Functional magnetic resonance imaging (fMRI), Eye tracking

## Abstract

**Background:**

Childhood disintegrative disorder (CDD) is a rare form of autism spectrum disorder (ASD) of unknown etiology. It is characterized by late-onset regression leading to significant intellectual disability (ID) and severe autism. Although there are phenotypic differences between CDD and other forms of ASD, it is unclear if there are neurobiological differences.

**Methods:**

We pursued a multidisciplinary study of CDD (*n* = 17) and three comparison groups: low-functioning ASD (*n* = 12), high-functioning ASD (*n* = 50), and typically developing (*n* = 26) individuals. We performed whole-exome sequencing (WES), copy number variant (CNV), and gene expression analyses of CDD and, on subsets of each cohort, non-sedated functional magnetic resonance imaging (fMRI) while viewing socioemotional (faces) and non-socioemotional (houses) stimuli and eye tracking while viewing emotional faces.

**Results:**

We observed potential differences between CDD and other forms of ASD. WES and CNV analyses identified one or more rare de novo, homozygous, and/or hemizygous (mother-to-son transmission on chrX) variants for most probands that were not shared by unaffected sibling controls. There were no clearly deleterious variants or highly recurrent candidate genes. Candidate genes that were found to be most conserved at variant position and most intolerant of variation, such as *TRRAP*, *ZNF236*, and *KIAA2018*, play a role or may be involved in transcription. Using the human BrainSpan transcriptome dataset, CDD candidate genes were found to be more highly expressed in non-neocortical regions than neocortical regions. This expression profile was similar to that of an independent cohort of ASD probands with regression. The non-neocortical regions overlapped with those identified by fMRI as abnormally hyperactive in response to viewing faces, such as the thalamus, cerebellum, caudate, and hippocampus. Eye-tracking analysis showed that, among individuals with ASD, subjects with CDD focused on eyes the most when shown pictures of faces.

**Conclusions:**

Given that cohort sizes were limited by the rarity of CDD, and the challenges of conducting non-sedated fMRI and eye tracking in subjects with ASD and significant ID, this is an exploratory study designed to investigate the neurobiological features of CDD. In addition to reporting the first multimodal analysis of CDD, a combination of fMRI and eye-tracking analyses are being presented for the first time for low-functioning individuals with ASD. Our results suggest differences between CDD and other forms of ASD on the neurobiological as well as clinical level.

**Electronic supplementary material:**

The online version of this article (doi:10.1186/s13229-017-0133-0) contains supplementary material, which is available to authorized users.

## Background

Autism spectrum disorder (ASD) is defined by deficits in social communication and interaction and restricted, repetitive patterns of behavior, interests, or activities [1]. Decades before Kanner published his landmark paper describing autism [2], Heller reported on six normally developing children who experienced a severe regression in skills between 3 and 4 years of age leading to global impairments, including autistic features [3, 4]. Heller termed the condition *dementia infantilis*, which was included in the ICD-10 [5] and DSM-IV [6] as childhood disintegrative disorder (CDD). CDD was defined by normal development for at least the first 2 years of life followed by regression before age 10 years in at least two of the following areas: (1) expressive or receptive language, (2) social skills or adaptive behavior, (3) bowel or bladder control, (4) play, and (5) motor skills. There has been much debate as to whether CDD is a late-onset variant of autism or a distinct entity [7, 8]. CDD was subsumed by the diagnosis ASD in the DSM-5 [1], since there was little scientific basis for including CDD as a separate disorder [9].

There are, however, important phenotypic differences between CDD and other forms of ASD [8, 10–15]. While symptoms of ASD are usually recognized by 2 years of age, the onset of symptoms in CDD is usually between 3 and 4 years of age. While approximately a third of children with ASD experience a regression in skills, again usually by age 2 years [16], CDD is defined by regression, which is characteristically of later onset, more global in extent, and more severe in degree. Indeed, children with CDD generally have the poorest outcome among individuals with ASD, usually with severe loss of cognitive and communication skills [8, 11]. In contrast to CDD, children who are diagnosed with ASD later than the typical age range tend to be higher functioning, leading to the delay in diagnosis, and early subtle abnormalities are often noted in retrospect [10, 12]. The majority of children with CDD experience a distinct prodrome characterized by bouts of anxiety and terror [3, 4, 8, 17]. No consistent medical, environmental, or psychosocial triggers have been associated with CDD [8].

Our overarching question is whether there are neurobiological features that distinguish CDD from other forms of ASD. The genetic basis, neuroimaging abnormalities, and social phenotype of ASD are being intensively studied, but no similarly comprehensive studies have been published examining CDD for two important reasons. First, CDD is rare. While the prevalence of ASD is reported to be 1/68 [18], the prevalence of CDD is estimated to be 1–2/100,000 [19]. Second, conducting experimental protocols such as non-sedated functional magnetic resonance imaging (fMRI) and eye tracking with low-functioning subjects is extremely challenging. To study CDD, we used a multidisciplinary approach encompassing: (1) expert clinical characterization; (2) the identification of candidate genes and gene expression analysis; (3) an analysis of brain function, via fMRI, in response to viewing socioemotional (fearful faces) and non-socioemotional (houses) stimuli; and (4) the precise quantification of the social behavioral phenotype using eye tracking. This study is novel not only in examining the neurobiological features of CDD but also in obtaining a combination of reliable non-sedated fMRI and eye-tracking data from low-functioning individuals on the autism spectrum.

## Methods

A detailed description of all methods can be found in Additional file 1: Supplementary information. We studied four cohorts: (1) subjects with CDD (*n =* 17, Table 1), (2) low-functioning [full-scale IQ (FSIQ) ≤ 75] subjects with ASD (LFASD, *n =* 12) and early-onset delays (<2 years old), (3) high-functioning (FSIQ ≥ 75) subjects with ASD (HFASD, *n =* 50) and early-onset delays (<2 years old), and (4) typically developing subjects (TD, *n =* 26). The genetics analysis focused on the CDD cohort whereas the fMRI and eye-tracking analyses included subsets of each cohort. We performed whole-exome sequencing (WES) and copy number variant (CNV) analyses of 15 families affected by CDD, which included 15 probands, 13 unaffected sibling controls, and their parents (Additional file 2: Table S1), to identify three types of rare [novel or found at most once across 1000 Genomes (May 2011 release), NHLBI GO ESP Exome Variant Server (ESP6500SI-V2), and in-house database of 2500 exomes] protein-changing variants: (1) de novo, (2) homozygous, and (3) hemizygous (mother-to-son transmission on chrX). We included one additional category for family CDD17 since the father and paternal grandfather reportedly have high-functioning autism: paternally inherited likely gene-disrupting (LGD) variants (premature stop codon, splice site disruption, deletion). We used the human BrainSpan exon-array transcriptome dataset [20] to plot the brain expression profile of CDD candidate genes and conduct co-expression analysis. To study neural systems, we used non-sedated fMRI, and a blocked design involving the presentation of grayscale fearful face (NimStim set of facial expressions) [21] and house (lab database) images to determine brain activation patterns across the four cohorts [CDD (*n =* 7), LFASD (*n =* 7), HFASD (*n =* 14), and TD (*n =* 19)]. To quantify the social phenotype of our four cohorts [CDD (*n =* 5), LFASD (*n =* 7), HFASD (*n =* 32), and TD (*n =* 14)], we collected eye-tracking data as they viewed emotional faces [21].

**Table 1:** CDD cohort: clinical characteristics and the modalities by which each proband was studied

Subject	Sex	Age at onset (months)	Anxiety prodrome	Multiple regressions	Length of regression (months)^a^	Level of ID	IQ	Cognitive/developmental test^b^	Language loss	Social or adaptive loss	Incontinence	Play loss	Motor skills loss	Seizures	1^0^ relative with ASD	Genetics	fMRI	ET	Age at fMRI, ET (months)
CDD03-03	M	30	No	No	5	Profound	18	Mullen	Yes	Yes	Yes	Yes	Yes	No	No	Yes	No	No	n/a
CDD05-03	M	37	Yes	No	9	Severe	16	Mullen	Yes	Yes	Yes	Yes	Yes	Yes	No	Yes	Yes	Yes	176
CDD07-03	M	45	Yes	No	4	Profound	9	Mullen	Yes	Yes	Yes	Yes	Yes	No	No	Yes	No	No	n/a
CDD08-03	M	80	Yes	Yes	3	Moderate	46	DAS	Yes	Yes	No	Yes	Yes	No	No	Yes	Yes	Yes	139, 154
CDD09-03	M	44	No	No	6	Mild	64	DAS	Yes	Yes	No	Yes	No	No	No	Yes	Yes	No	52
CDD10-03	M	38	Yes	No	3	Profound	19	Mullen	Yes	Yes	Yes	Yes	Yes	No	No	Yes	No	Yes	122
CDD11-03	M	40	Yes	No	4	Profound	8	Mullen	Yes	Yes	No	Yes	Yes	No	No	Yes	Yes	No	206
CDD12-03	F	36	Yes	No	3	Severe	33	Mullen	Yes	Yes	Yes	Yes	Yes	No	No	Yes	No	Yes	87
CDD13-03	F	31	No	No	80	Profound	18	Mullen	Yes	Yes	Yes	Yes	Yes	No	MZ twin (CDD)^c^	Yes	Yes	Yes	92
CDD13-04	F	28	No	Yes	4	Profound	16	Mullen	Yes	Yes	Yes	Yes	Yes	No	MZ twin (CDD)^c^	Yes	Yes	No	92
CDD15-03	M	84	Yes	Yes	2	Severe	n/a	n/a	Yes	Yes	Yes	Yes	No	No	No	Yes	No	No	n/a
CDD16-03	M	53	Yes	Yes	6	Moderate	n/a	n/a	Yes	Yes	Yes	Yes	No	No	Brother (ASD)	Yes	No	No	n/a
CDD17-03	M	68	Yes	No	3	Moderate	44	DAS	Yes	Yes	No	Yes	No	No	Father (autistic features)	Yes	No	No	n/a
CDD19-03	M	31	No	No	24	Severe	26	Mullen	Yes	Yes	No	Yes	No	No	Brother (autistic features)	No	Yes	No	60
CDD20-03	M	51	Yes	Yes	9	Mild	74	DAS	Yes	Yes	Yes	Yes	No	No	MZ twin (ASD)	Yes	No	No	n/a
CDD21-03	F	30	Yes	No	5	Severe	30	Mullen	Yes	Yes	No	Yes	Yes	No	No	Yes	No	No	n/a
CDD22-03	M	48	Yes	No	4	Severe	30	Leiter	Yes	Yes	Yes	Yes	Yes	No	No	Yes	No	No	n/a

*Abbreviations*: *ASD* autism spectrum disorder, *CDD* childhood disintegrative disorder, *DAS* Differential Ability Scales, *ET* eye tracking, *F* female, *fMRI* functional magnetic resonance imaging, *ID* intellectual disability, *IQ* intelligence quotient, *M* male, *MZ* monozygotic

^a^If multiple regressions occurred, the number indicates the length of the first period of regression

^b^For subjects who received the Mullen Scales, their full-scale IQ is based on a ratio IQ

^c^Subjects CDD13-03 and 13-04 are monozygotic twin girls with CDD who are counted as one proband for the genetics studies

## Results

### Study subjects

Clinical characteristics of the CDD cohort and the number of subjects examined by each study modality are shown in Table 1. The sex ratio of 3.25 males to 1 female is similar to that reported for ASD. The mean and median age at onset of symptoms was 46 and 40 months, respectively, with a range of 28 to 84 months. Seventy percent of subjects experienced a prodrome of anxiety and terror. Thirty percent of subjects had multiple episodes of regression. The length of the first regressive episode ranged from 2 months to almost 7 years in one subject. Most subjects have severe to profound ID, with the mean and median IQ being 30 and 26, respectively, with a range of 8 to 74. All had loss of language skills, loss of social skills or adaptive behavior, and loss of play skills. Sixty-five percent had loss of bowel or bladder control, and the same proportion had loss of motor skills. Although CDD has been reported to be almost always sporadic, a few of our subjects have immediate family members with ASD or autistic features, including two sets of monozygotic twins. Both members of one pair (CDD13-03/04) have CDD; in the other pair (CDD20-03/04), one has CDD and the other has ASD.

### Genetics

Given the rarity, severity, and apparently sporadic transmission seen in most CDD cases, we hypothesized that rare variants of large effect contribute to the etiology. Indeed, there is abundant evidence for the contribution of rare variants to ASD [22–24]. As shown in Table 2, we found one or more rare variants for all but one proband, which were not shared by any unaffected sibling controls (Additional file 2: Table S2). We also looked for compound heterozygous variants in subjects by searching for additional variants in genes affected by de novo variants but did not find any. The rates of all high-probability (Bayesian quality score ≥ 50) de novo variants were 0.80/proband exome and 0.92/sibling exome (Additional file 2: Table S3), which are similar to the overall rates calculated from 11 recent WES studies of neurodevelopmental disorders: 1.00/proband exome, *n* = 2358; 0.82/control exome, *n* = 731 [25]. There were no significant differences in the rates of non-synonymous de novo, homozygous, and hemizygous variants (Additional file 2: Table S3); the rate of brain-expressed genes affected; phylogenetic *P* value (PhyloP) conservation scores at variant positions; Residual Variation Intolerance Scores (RVIS); and polymorphism phenotyping v2 (PolyPhen-2) scores (Additional file 2: Table S2) between the probands and siblings.

**Table 2:** Rare variants unique to CDD probands

Sex	Inheritance	Gene	AA change	Genomic coordinate (hg19)	Reference	Variant	Brain exp	PhyloP^b^	RVIS	PolyPhen2	Gene-associated OMIM disorder (#, inheritance)	Variants identified in ID, ASD, EE, SCZ, DD: #, inheritance, variant type [reference]
CDD03-03												
M	Hemizygous	*NRK*	K336R	chrX:105150568	A	G	Yes	5.88	n/a	0.999 (Mis3)	n/a	ID: 1 DN, 7 n/a missense [42–44]
M	Hemizygous	*TBC1D8B*	L653I	chrX:106093374	C	A	Yes	4.84	-1.15	0.999 (Mis3)	n/a	ASD: 1 DN missense [23]; ID: 1 DN, 2 n/a missense [42, 43]
M	Hemizygous	*NKRF*	K50R	chrX:118725239	T	C	Yes	1.39	-0.54	0.005 (Mis1)	n/a	ID: 3 n/a missense [44]
M	Hemizygous	*SAGE1*	A362V	chrX:134989926	C	T	Yes	-0.03	0.01	0.000 (Mis1)	n/a	ASD: 1 DN missense [23]; ID: 4 n/a missense [42, 44]
CDD05-03												
M	Homozygous	*TCTEX1D2*	R110C	chr3:196022930	G	A	Yes	2.69	0.28	1.000 (Mis3)	n/a	SCZ: 1 DN, 20 n/a deletion [45, 46]
M	Hemizygous	*USP26*	P683L	chrX:132160201	G	A	No	-1.09	0.46	0.000 (Mis1)	n/a	ID: 4 n/a missense [42, 44]
CDD07-03												
M	De novo	*BBS9*	L376P	chr7:33376163	T	C	Yes	5.72	1.05	0.222 (Mis1)	Bardet-Biedl syndrome 9 (615986, AR)	Syndromic ID
M	De novo	*TRRAP*	P1781S	chr7:98548580	C	T	Yes	7.70	-6.14	1.000 (Mis3)	n/a	ASD: 3 DN missense [23]; EE: 1 DN missense [47]; SCZ: 1 DN missense, 1 DN SS [48]; DD: 3 DN missense [49]
M	Homozygous	*DNMT3B*	A364T	chr20:31383238	G	A	Yes	0.31	-1.70	0.000 (Mis1)	Immunodeficiency-centromeric instability-facial anomalies syndrome 1 (242860, AR)	Syndromic ID
M	Hemizygous	*CDR1*	G161R	chrX:139866051	C	T	Yes	0.37	0.15	0.995 (Mis3)	n/a	ID: 2 n/a missense [42]
M	Hemizygous	*FAM50A*	E136K	chrX:153674872	G	A	Yes	6.75	-0.01	0.010 (Mis1)	n/a	ID: 1 n/a missense [42]; EE: 1 DN missense [47]
CDD08-03												
M	Hemizygous	*CNGA2*	F385S	chrX:150912129	T	C	No	7.56	0.49	0.999 (Mis3)	n/a	ID: 5 n/a missense [42, 44]
CDD09-03												
M	Homozygous	*NOP9*	A161_E162insEE	chr14:24769850	*	I:AGGAGG	Yes	0.59	0.98	n/a	n/a	DD: 1 DN indel [49]
M	Hemizygous	*ZXDA*	V264L	chrX:57936065	C	A	Yes	-0.44	n/a	0.000 (Mis1)	n/a	ID: 1 DN, 1 n/a missense [42, 43]
M	Hemizygous	*SPANXN2*	L41F	chrX:142795555	C	A	No	-1.83	0.41	0.946 (Mis2)	n/a	ID: 1 n/a missense [42]
CDD10-03												
M	De novo	*NAV2*	R2046W	chr11:20122629	C	T	Yes	3.78	-1.56	1.000 (Mis3)	n/a	ASD: 2 DN missense [23]
CDD12-03												
F	Homozygous	*ADAMTS18*	S660N	chr16:77359816	C	T	Yes	4.90	-0.09	0.001 (Mis1)	Microcornea, myopic chorioretinal atrophy, and telecanthus (615458, AR)	
CDD13-03 and 04 (MZ twins)^a^												
F	De novo	*BBS5*	I76M	chr2:170344335	A	G	Yes	1.29	0.04	0.82 (Mis2)	Bardet-Biedl syndrome 5 (615983, AR)	Syndromic ID
F	De novo	*NSD1*	R964W	chr5:176639097	C	T	Yes	0.27	-1.55	0.997 (Mis3)	Sotos syndrome 1 (117550, AD), Beckwith-Wiedemann syndrome (130650, AD)	Syndromic ID and ASD
F	De novo	*OGDHL*	3′UTR deletion	chr10:50940935-50943068	*	CNV: deletion	Yes	5.43	-0.54	n/a	n/a	
F	Hemizygous	*TUBGCP5*	T274S	chr15:22846945	A	T (paternal allele deleted)	Yes	8.29	-0.77	0.891 (Mis2)	n/a	ASD: 1 DN missense [23]; ID: 1 inh deletion [50]; EE: 1 inh deletion [50]; SCZ: 1 inh deletion [50]
CDD15-03												
M	De novo	*ZNF236*	L1599H	chr18:74659496	T	A	Yes	5.43	-1.53	0.999 (Mis3)	n/a	
M	Hemizygous	*SUPT20HL2*	G472R	chrX:24330019	C	T	No	1.32	n/a	0.884 (Mis2)	n/a	
CDD16-03												
M	Homozygous	*PRKCSH*	E314_E315del	chr19:11558343-11558348	*	D:GGAGGA	Yes	1.61	-0.89	n/a	Polycystic liver disease 1 (174050, AD)	
M	Hemizygous	*SUPT20HL2*	P795A	chrX:24329050	G	C	No	-0.55	n/a	0.047 (Mis1)	n/a	
M	Hemizygous	*USP9X*	A843G	chrX:41027363	C	G	Yes	7.62	-1.62	0.102 (Mis1)	Mental retardation, X-linked 99 (300919, XLR and 300968, XLD)	Syndromic ID and EE
M	Hemizygous	*GATA1*	A55T	chrX:48649679	G	A	Yes	1.06	-0.60	0.993 (Mis3)	Anemia (300835, XLR); Thrombocytopenia (314050, XLR and 300367, XLR)	
M	Hemizygous	*MTMR8*	N265K	chrX:63564995	G	T	Yes	4.00	-0.77	1.000 (Mis3)	n/a	ASD: 1 DN missense [23], 1 hemizygous SS [51]; ID: 5 n/a missense [42, 44]
M	Hemizygous	*DCX*	M51V	chrX:110654052	T	C	Yes	0.66	-0.10	0.002 (Mis1)	Lissencephaly, X-linked (300067, XL)	Syndromic ID and EE
M	Hemizygous	*CT47B1*	L236P	chrX:120008818	A	G	No	-1.15	n/a	0.001 (Mis1)	n/a	ASD: 1 DN missense [23]; ID: 2 n/a missense [44]
CDD17-03												
M	Heterozygous	*CLUL1*	5′SS disruption	chr18:618107	G	A (paternal)	Yes	6.04	0.66	n/a	n/a	
M	Hemizygous	*BCOR*	F1023L	chrX:39930395	G	C	Yes	8.97	-3.03	0.999 (Mis3)	Microphthalmia, syndromic 2 (300166, XLD)	Syndromic ID
M	Hemizygous	*BEX2*	Y97C	chrX:102564711	T	C	Yes	0.78	0.64	0.046 (Mis1)	n/a	ASD: 1 n/a missense [52]; ID: 1 n/a missense [42]; SCZ: 1 n/a missense [52]
M	Hemizygous	*VSIG1*	Q88R	chrX:107304707	A	G	Yes	1.19	-0.18	0.856 (Mis2)	n/a	ASD: 2 hemizygous SS [51]; ID: 1 n/a missense [44]
M	Hemizygous	*SRPK3*	R619Q	chrX:153050929	G	A	Yes	7.69	-0.33	1.000 (Mis3)	n/a	ID: 7 n/a missense [42, 44, 53]
M	Hemizygous	*USP9Y*	R122X	chrY:14837084	C	T	Yes	2.63	n/a	n/a	Spermatogenic failure, Y-linked, 2 (415000, YL)	
CDD20-03^a^												
M	Homozygous	*KIAA2018*	Q1472del	chr3:113376112-113376114	*	D:GCT	Yes	3.58	-0.71	n/a	n/a	ASD: 1 DN nonsense, 4 n/a missense [22]; EE: 1 DN missense [47]; SCZ: 1 DN missense [54]
M	Homozygous	*BRIP1*	V193I	chr17:59924512	C	T	Yes	-0.69	-0.64	0.000 (Mis1)	Breast cancer, early-onset (114480, AD); Fanconi anemia, complementation group J (609054, AR)	
M	Hemizygous	*PDK3*	K410Q	chrX:24557261	A	C	Yes	2.44	0.13	0.001 (Mis1)	Charcot-Marie-Tooth disease, 6 (300905, XLD)	
M	Hemizygous	*ARSF*	R386Q	chrX:3021857	G	A	Yes	6.00	0.36	0.999 (Mis3)	n/a	ASD: 1 n/a nonsense [51]; ID: 4 n/a missense, 3 n/a indel, 2 n/a SS [42, 44]
M	Hemizygous	*ALAS2*	V193I	chrX:55047546	C	T	Yes	3.26	0.13	0.803 (Mis2)	Anemia, sideroblastic, 1 (300751, XLR); Protoporphyria, erythropoietic (300752, XLD)	
M	Hemizygous	*STARD8*	3 bp deletion	chrX:67938303-67938305	*	D:AAG	Yes	3.59	0.74	n/a	n/a	ID: 2 n/a missense, 1 n/a indel [42, 44]
M	Hemizygous	*CXorf57*	R545T	chrX:105882817	G	C	Yes	6.24	-0.40	1.000 (Mis3)	n/a	ID: 2 n/a missense [42, 44]
M	Hemizygous	*ALG13*	S891F	chrX:110980084	C	T	Yes	4.77	-1.00	0.986 (Mis3)	Epileptic encephalopathy, early infantile, 36 (300884, XLD)	Syndromic ID and EE
CDD21-03												
F	Homozygous	*RAD51C*	T287A	chr17:56798128	A	G	Yes	5.16	0.11	0.988 (Mis3)	Fanconi anemia, complementation group O (613390, AR)	
F	Homozygous	*SHANK3*	G277R	chr22:51117800	G	A	Yes	1.95	n/a	0.002 (Mis1)	Phelan-McDermid syndrome (606232, DN); Schizophrenia 15 (613950, DN)	Syndromic ID, ASD, EE, SCZ
CDD22-03												
M	Homozygous	*DAP3*	G5E	chr1:155679584	G	A	Yes	1.86	-0.22	0.890 (Mis2)	n/a	ASD: 1 DN missense [23]
M	Hemizygous	*ENOX2*	E12K	chrX:129843232	C	T	Yes	-0.35	-0.43	0.000 (Mis1)	n/a	ID: 1 hemizygous, 1 n/a missense [42, 55]

*Abbreviations*: *AA* amino acid, *AD* autosomal dominant, *AR* autosomal recessive, *ASD* autism spectrum disorder, *CDD* childhood disintegrative disorder, *CNV* copy number variant, *DD* developmental disorders, *DN* de novo, *EE* epilepsy/epileptic encephalopathy, *F* female, *indel* insertion/deletion, *inh* inherited, *ID* intellectual disability, *M* male, *Mis1* Missense1 (benign), *Mis2* Missense2 (possibly damaging), *Mis3* Missense3 (probably damaging), *MZ* monozygotic, *OMIM* Online Mendelian Inheritance in Man, *PhyloP* phylogenetic P-value, *PolyPhen-2* polymorphism phenotyping v2, *RVIS* Residual Variation Intolerance Score, *SCZ* schizophrenia/childhood-onset schizophrenia, *SS* splice site, *XL* X-linked, *XLD* X-linked dominant, *XLR* X-linked recessive, *YL* Y-linked

^a^No discordant variants were confirmed between monozygotic twin girls CDD13-03 and 04 (CDD) and between monozygotic twin boys CDD20-03 (CDD) and 04 (ASD)

^b^PhyloP scores for indels and CNVs were calculated by averaging the PhyloP scores for all positions affected

*wild-type sequence

We found one de novo genic CNV in a proband (0.07/proband, Table 2), which is similar to rates previously reported for ASD [26], and none in siblings. The proband CNV is a 2 kb heterozygous deletion of the 3′UTR of *OGDHL*, which encodes a component of a mitochondrial protein complex implicated in neurodegeneration [27]. One gene, *SUPT20HL2*, and two gene families, *USP* and *BBS*, are affected in more than one CDD proband. Two hemizygous missense variants were identified in *SUPT20HL2*, which encodes a putative transcription factor but could be a pseudogene according to the UniProtKB database (http://www.uniprot.org/). Three members of the *USP* (*ubiquitin*-*specific peptidase*) gene family are affected in CDD probands: *USP9X* (hemizygous missense), *USP9Y* (paternally-inherited non-sense), and *USP26* (hemizygous missense). They encode deubiquitinating enzymes that prevent the degradation of proteins. Two members of the Bardet-Biedel Syndrome (BBS) gene family, which is involved in ciliogenesis, have de novo missense variants in CDD probands: *BBS5* and *BBS9*. Although the specific protein-changing variants identified in CDD subjects were rare and not previously associated with disease, we reviewed the literature and found some overlap between CDD candidate genes and genes potentially associated with other neurological disorders (Table 2).

There were no clearly deleterious variants in the CDD probands. To identify potentially pathogenic variants, we considered a combination of factors: (1) positive brain expression, (2) PhyloP score ≥ 1.30 (*P* = 0.05 for conservation), (3) negative RVIS (gene intolerant of variation), and (4) PolyPhen-2 classification of probably damaging missense (or n/a due to a variant other than missense). Of the 47 CDD candidate genes, 14 met all of these criteria: *NRK*, *TBC1D8B*, *TRRAP*, *NAV2*, *OGDHL*, *ZNF236*, *PRKCSH*, *MTMR8*, *BCOR*, *SRPK3*, *USP9Y*, *KIAA2018*, *CXorf57*, *and ALG13* (Table 2)*.* To further refine this list, inspection of sequencing data from the Exome Aggregation Consortium (http://exac.broadinstitute.org/) revealed that: (1) the variants in all of the genes except *NAV2*, *MTMR8*, and *ALG13* are novel or found at most once in the dataset and (2) among the remaining 11 genes, 4 are among the 5% most intolerant: *TRRAP*, *ZNF236*, *BCOR*, and *KIAA2018*.

*TRRAP* (*transformation/transcription domain-associated protein*)affected by a de novo missense variant in a male CDD proband; it encodes a component of histone acetyltransferase complexes and is involved in DNA transcription and repair. It is not associated with an OMIM disorder, but de novo variants have been identified in other neurological disorders (Table 2). *ZNF236* (*Zinc Finger Protein 236*) is also affected by a de novo missense variant in a male proband; it may be involved in transcriptional regulation (UniProtKB) but is not associated with a known disorder. *BCOR* (*BCL6 Corepressor*) is affected by a hemizygous missense variant in a male CDD proband; it encodes a transcriptional corepressor. It is associated with syndromic microphthalmia, which can have the feature of ID but otherwise does not characterize the CDD proband and is usually caused by truncating mutations in females. *KIAA2018* is affected by a homozygous one-amino acid deletion in a male CDD proband; it is also known as USF3 (*upstream transcription factor 3*). It is not associated with an OMIM disorder, but de novo variants have been identified in other neurological disorders (Table 2). Of note, all of these top candidate genes either play a role or may be involved in transcription, which characterizes many ASD-associated genes as well [22].

**Fig. 1:**
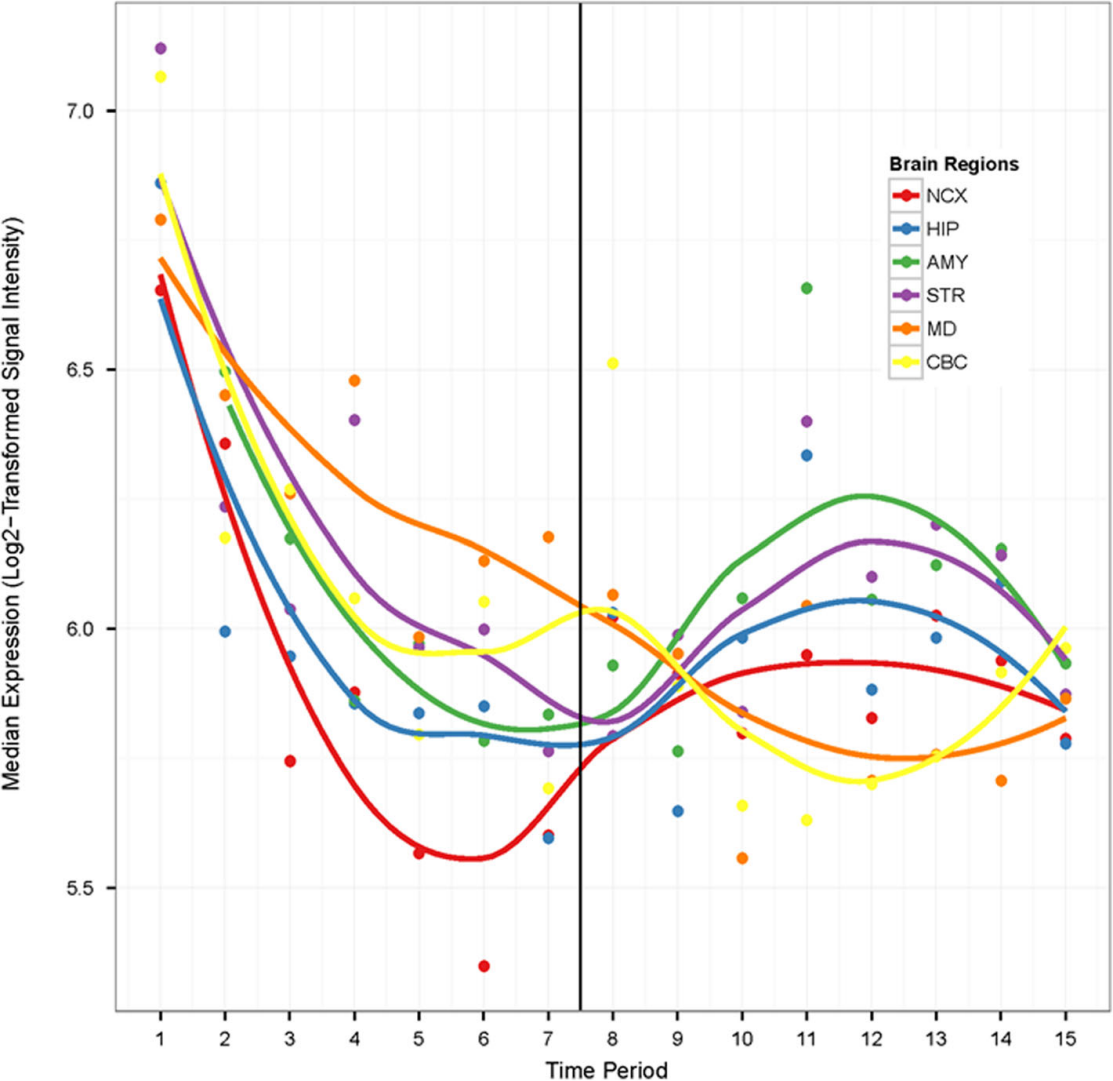
Median expression levels of CDD candidate genes (*n* = 40) by brain region and time period (Additional file 2: Table S5) using the human BrainSpan exon-array transcriptome dataset [20]. The dark vertical line indicates birth. Log_2_-transformed signal intensity ≥ 6 in at least one sample is considered positive expression [20]. *AMY* amygdala, *CBC* cerebellar cortex, *HIP* hippocampus *MD* mediodorsal nucleus of the thalamus, *NCX* neocortex, *STR* striatum

Using the human BrainSpan exon-array transcriptome dataset [20], we plotted the median expression level of the CDD candidate genes as a group for all the brain regions available from embryonic to late adulthood stages (*n* = 40 genes represented once in the core probe set, Additional file 2: Table S4). As shown by the expression profile in Fig. 1, CDD candidate genes are more highly expressed in non-neocortical regions [hippocampus (HIP), amygdala (AMY), striatum (STR), mediodorsal nucleus of the thalamus (MD), and/or cerebellar cortex (CBC)] compared to neocortical regions across the lifespan (Additional file 2: Table S5). Moreover, there are increasing levels of expression in the AMY, STR, and HIP during periods 10 (1–6 years old) and 11 (6–12 years-old), the range that encompasses the age of onset of symptoms in our CDD cohort.

**Fig. 2:**
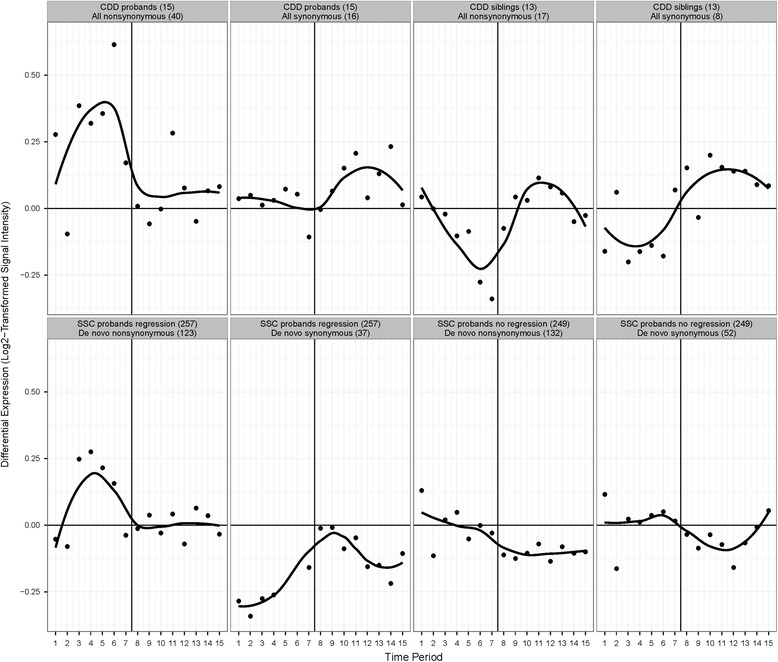
Differential expression levels of various gene sets. The difference in median expression levels (non-neocortical minus neocortical brain regions) is shown for genes affected by non-synonymous or synonymous variants in CDD probands, their unaffected siblings, SSC probands with regression, and SSC probands without regression. The number in parentheses indicates the number of subjects or variants, and the *dark vertical line* in each panel indicates birth. For potential CDD candidate genes, the difference reaches a maximum positive value at period six (mid-fetal stages); significance was confirmed by permutation testing with 100,000 iterations of 40 randomly selected genes (*P* = 0.0022). *CDD* childhood disintegrative disorder, *SSC* Simons Simplex Collection

Given this observation, we compared the difference in median expression levels between non-neocortical and neocortical regions for genes affected by non-synonymous and synonymous variants in CDD probands, their unaffected siblings, and ASD probands from the Simons Simplex Collection (SSC) with and without regression [23] matched by sex, age at evaluation, IQ, and autism symptom severity (see Additional file 1: Supplementary information for cohort selection details). The expression profile of CDD candidate genes is qualitatively distinct from the other gene sets, except that it is similar to the profile of genes affected by non-synonymous variants in SSC probands with regression, even though they have only one gene, *NAV2*, in common (Fig. 2, Additional file 2 : Tables S4 and S6). The difference in expression, non-neocortical minus neocortical, reaches a maximum positive value at mid-fetal stages. For CDD candidate genes, this occurs at period six [19–24 postconceptual weeks (PCW)]; permutation testing with 100,000 iterations of 40 randomly selected genes from the BrainSpan dataset confirmed the significance of this differential expression (*P =* 0.0022). We extended the analysis to several other gene sets, such as those identified in SSC probands and unaffected siblings with non-synonymous, synonymous, and LGD variants; genes most significantly associated with ASD by three recent large WES and CNV studies [22–24]; and all genes in the BrainSpan dataset. The expression profile of genes affected by non-synonymous variants in CDD probands and SSC probands with regression is qualitatively distinct from these other sets as well (Additional file 1: Figure S1, Additional file 2: Tables S4 and S6).

**Fig. 3:**
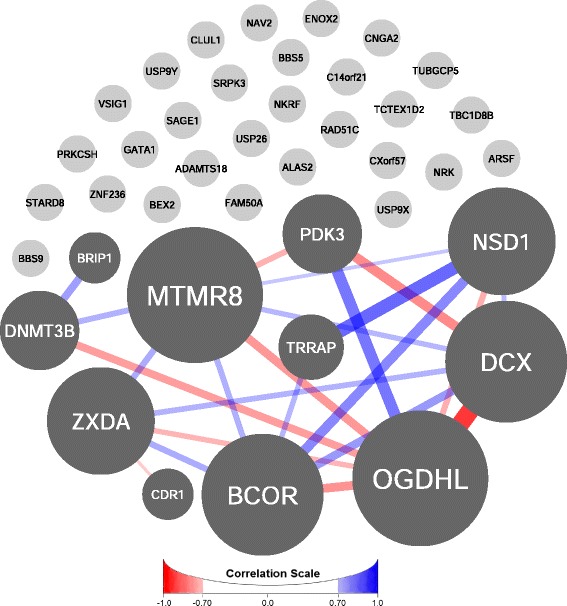
Gene coexpression network analysis. Eleven of the 40 CDD candidate genes are coexpressed with at least one other candidate gene across all brain regions and time periods with a Pearson correlation coefficient *r* ≥ 0.7 (Additional file 2: Table S7), a mean of 2.09 correlations/gene (*P* = 0.036), and a mean coefficient of 0.779 (*P* = 0.019, permutation testing with 100,000 iterations of 40 randomly selected genes). Positive correlations are shown in *blue*, and negative correlations are shown in *red*. The greater the magnitude of the coefficient, the wider and darker are the edges. The size of a node is proportional to the number of edges the node has

We also investigated whether CDD candidate genes are coexpressed with each other. Of the 40 candidate genes, 11 are coexpressed with at least one other candidate gene across all brain regions and time periods with a Pearson correlation coefficient *r* ≥ 0.7 (Fig. 3, Additional file 2: Table S7). There are 23 such connections, for a mean of 2.09 correlations/gene and a mean coefficient of 0.779. Permutation testing with 100,000 iterations of 40 randomly selected genes from the BrainSpan dataset revealed that observing 11 genes with at least 2.09 correlations/gene is significant (*P* = 0.036), as is observing 11 genes with a mean correlation coefficient of at least 0.779 (*P* = 0.019). Meeting both thresholds is also significant (*P* = 0.0059). Since all 11 CDD candidate genes which are coexpressed with each other have positive brain expression as per the BrainSpan dataset, permutation testing with 100,000 iterations was also performed with 40 randomly selected brain-expressed genes from BrainSpan. While observing 11 genes with at least 2.09 correlations/gene is not significant (*P* = 0.066), observing 11 genes with a mean correlation coefficient of at least 0.779 is significant (*P* = 0.022) as is meeting both thresholds (*P* = 0.011). Comparing the set of 11 coexpressed CDD candidate genes with the remaining set of 29 which are not coexpressed revealed no significant differences between the rate of brain-expressed genes, PhyloP scores, or PolyPhen-2 scores; however, the coexpressed genes are significantly more intolerant of variation (average RVIS −1.42 versus −0.15, *t*(35) = −2.91, *P* = 0.0062, independent *t* test, two-tailed). Gene ontology enrichment analysis using the Database for Annotation, Visualization and Integrated Discovery v6.8 (https://david.ncifcrf.gov/) for the whole set of CDD candidate genes, and the subset of 11 coexpressed genes did not identify significant enrichment of GO terms after Benjamini-Hochberg correction of *P* values.

### Neural systems

Given the universality of social deficits in ASD, dysfunction in brain systems subserving social perception, including the perception of faces, is a key focus of ASD research. Face and house visual stimuli reliably activate and dissociate systems involved in socioemotional (fearful faces) and non-socioemotional (houses) information processing. We studied four cohorts: CDD (*n =* 7), LFASD (*n =* 7), HFASD (*n =* 14), and TD (*n =* 19). Even though individuals with LFASD are more numerous than those with CDD, our sample size was still limited by the difficulty of obtaining high-quality neuroimaging (and eye-tracking) data in low-functioning subjects. That being said, to our knowledge, this is the first ever presentation of non-sedated fMRI data from individuals with ASD and marked ID.

**Fig. 4:**
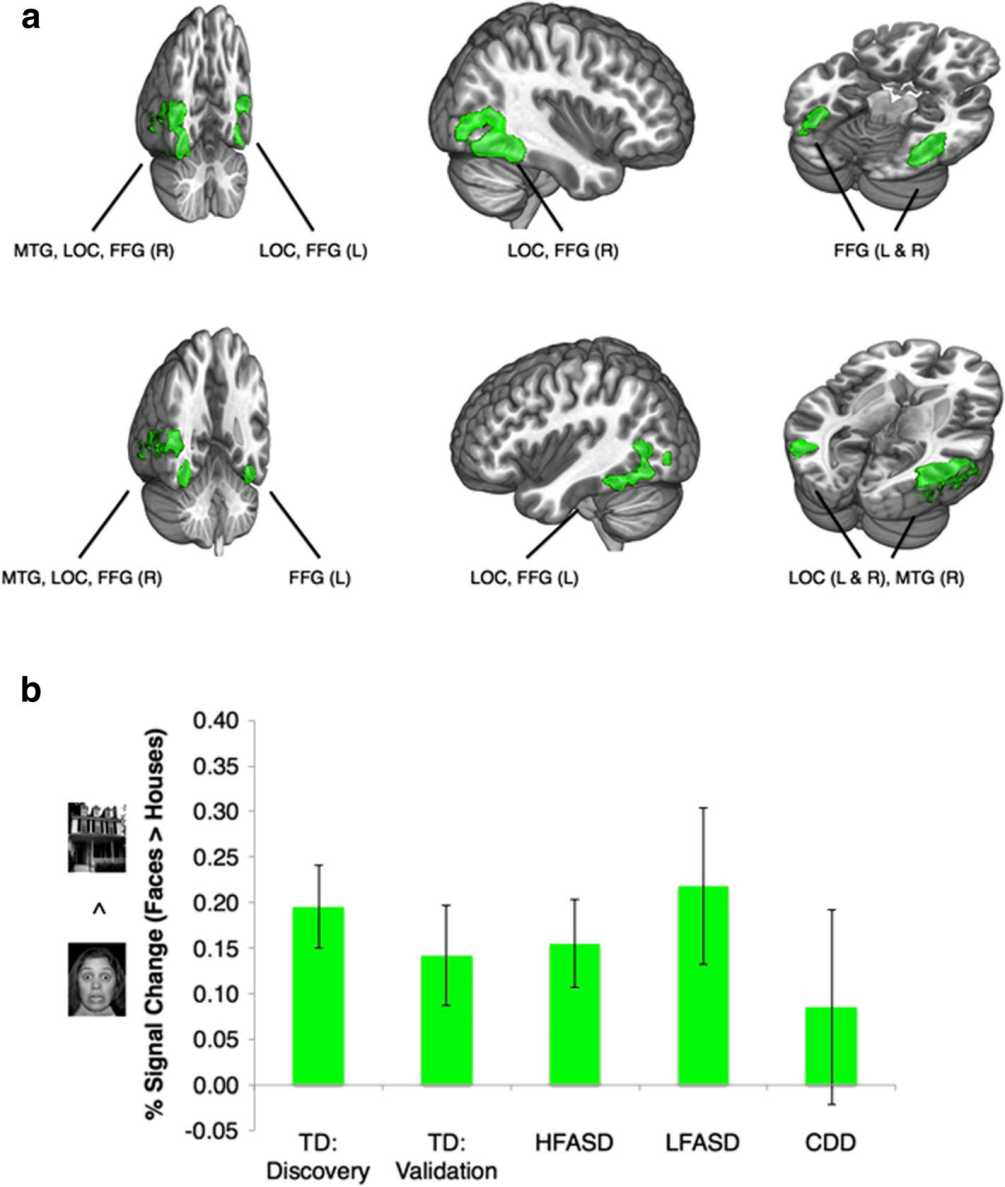
Brain regions of interest (ROIs) involved in processing socioemotional (*fearful face*) versus non-socioemotional (*house*) visual stimuli. **a** The *green* color brain map indicates regions of significant faces > houses activation in a discovery sample of 12 TD subjects (*Z* > 3.09, whole-brain corrected at the cluster-level *P* < 0.05). **b** These independently defined ROIs were then utilized for comparisons across the four remaining cohorts, a TD:validation sample (*n* = 7), HFASD (*n* = 14), LFASD (*n* = 7), and CDD (*n* = 7). The *bar graph* indicates the mean % signal change (faces > houses) for each cohort. Group differences were not significant when comparing the TD:validation and HFASD groups [*t*(19) = 0.17, *P* = 0.87, Cohen’s *d* = 0.08] and when comparing the LFASD and CDD groups [*t*(12) = 0.97, *P* = 0.35, Cohen’s *d* = 0.56]. The faces > houses response within the CDD group was not significantly greater than zero [*t*(6) = 0.80, *P* = 0.45, Cohen’s *d* = 0.30]. *Error bars* indicate standard error of the mean. All *P* values were calculated by independent *t* test and are two-tailed. *FFG* fusiform gyrus, *L* left, *LOC* lateral occipital cortex, *MTG* middle temporal gyrus, *R* right

There were no significant differences in sex, age, intracranial volume, and head movement in the scanner between the four cohorts. The CDD and LFASD groups were also not significantly different by IQ and autism severity, and the HFASD and TD groups were not significantly different by IQ (Additional file 2: Tables S8 and S9). First, we utilized a discovery sample of 12 of our 19 TD subjects in a whole-brain analysis for an independent localization of regions of interest involved in processing faces relative to houses. Figure 4a illustrates regions of ventrolateral occipitotemporal cortex where TD subjects exhibited significant faces > houses activation (Additional file 2: Table S10). These regions included the expected locations of well-known nodes of the occipitotemporal face-sensitive network including the fusiform face area [28, 29] and the occipital face area [30]. As shown in Fig. 4b and Additional file 2: Table S11, extraction of the mean percent signal change (faces > houses) for each of the four groups [TD:validation (the remaining 7 of the 19 TD subjects), HFASD, LFASD, and CDD] indicated an absence of group differences in the response to faces versus houses in these independently defined regions of interest when comparing the TD:validation and HFASD groups [*t*(19) = 0.17, *P* = 0.87, Cohen’s *d* = 0.08] and when comparing the LFASD and CDD groups [*t*(12) = 0.97, *P* = 0.35, Cohen’s *d* = 0.56]. The faces > houses response within the CDD group was not significantly greater than zero [*t*(6) = 0.80, *P* = 0.45, Cohen’s *d* = 0.30], suggesting an overall lack of sensitivity to faces in the occipitotemporal face-sensitive network as a whole. There is a well-established finding of hypoactivation to faces (versus houses) in the right, middle fusiform gyrus in HFASD relative to TD [31]. We were able to replicate this finding in our cohorts [*t*(31) = 3.54, *P* = 0.0013, Cohen’s *d* = 1.29]. However, comparison of faces > houses activity in CDD relative to TD revealed no significant difference [*t*(24) = 1.18, *P* = 0.25, Cohen’s *d* = 0.54], as did the comparison of the LFASD and TD groups [*t*(24) = 1.10, *P* = 0.28, Cohen’s *d* = 0.51] (Additional file 1: Figure S2 and Additional file 2: Table S12).

**Fig. 5:**
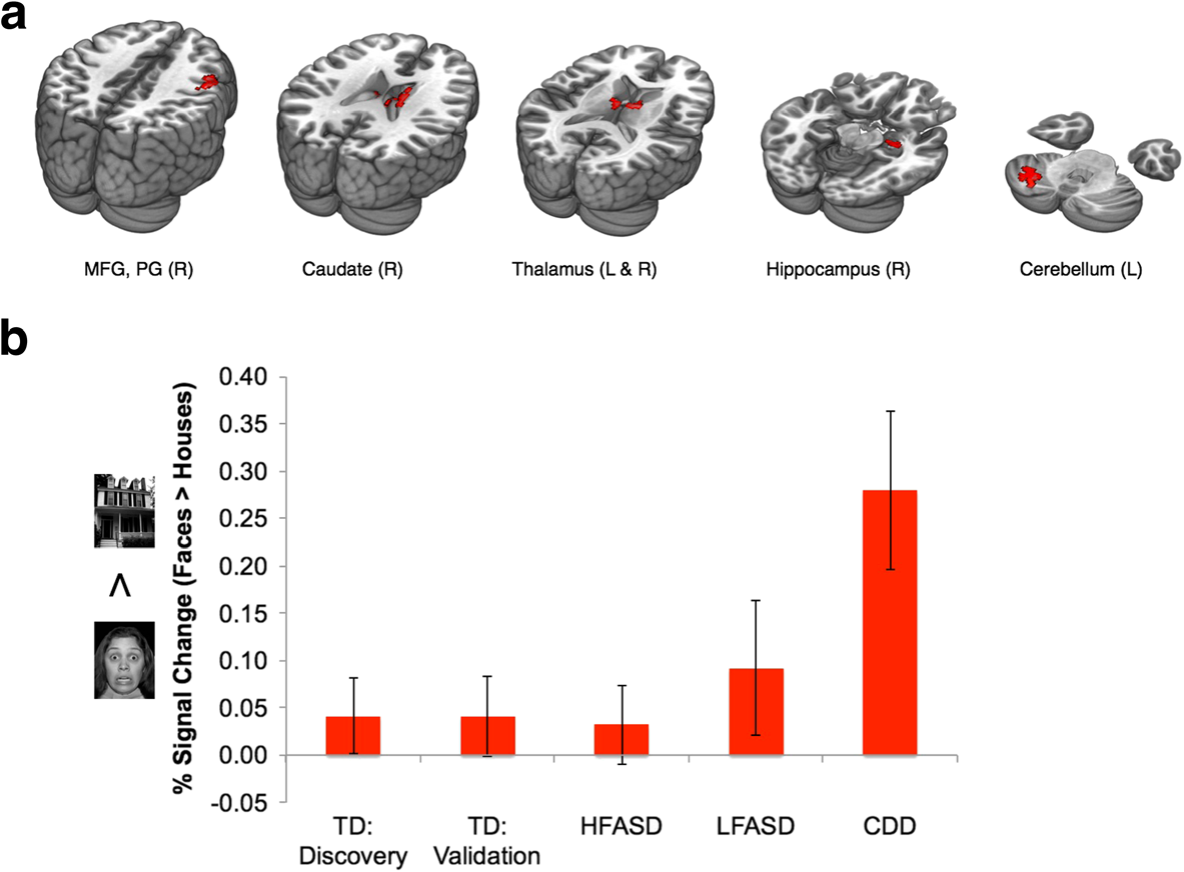
CDD whole-brain fMRI analysis. **a** The *red color* brain map indicates regions of significant faces > houses activation in the CDD subjects (*Z* > 3.09, whole-brain corrected at the cluster-level *P* < 0.05). **b** The *bar graph* indicates the mean % signal change (faces > houses) within these areas for each cohort: TD:discovery (*n* = 12), TD:validation (*n* = 7), HFASD (*n* = 14), LFASD (*n* = 7), and CDD (*n* = 7). The CDD cohort differed significantly from HFASD [*t*(19) = 2.98, *P* = 0.0076, Cohen’s *d* = 1.45] but not from LFASD [*t*(12) = 1.71, *P* = 0.11, Cohen’s *d* = 0.99]. *Error bars* indicate standard error of the mean. All *P* values were calculated by independent *t* test and are two-tailed. *MFG* middle frontal gyrus, *PG* precentral gyrus

Given the possible lack of sensitivity to faces in the ventrolateral occipitotemporal cortex in CDD, we next conducted a whole-brain evaluation of the CDD subjects to localize the neuroanatomical substrates of face perception in these individuals. As illustrated in Fig. 5a, CDD subjects exhibited faces > houses activity in the middle frontal gyrus, precentral gyrus, caudate (striatum), thalamus, hippocampus, and cerebellum (Additional file 2: Table S13). These overlap with brain regions determined to have the highest levels of CDD candidate gene expression (Fig. 1). As shown in Fig. 5b and Additional file 2: Table S14, comparison of the mean percent signal change (faces > houses) from these regions of interest revealed a significant difference between CDD and HFASD [*t*(19) = 2.98, *P* = 0.0076, Cohen’s *d* = 1.45], but no significant difference between CDD and LFASD [*t*(12) = 1.71, *P* = 0.11, Cohen’s *d* = 0.99]. The LFASD group showed an intermediate phenotype to that of HFASD and CDD groups (Fig. 5b).

### Eye-gaze behavior

**Fig. 6:**
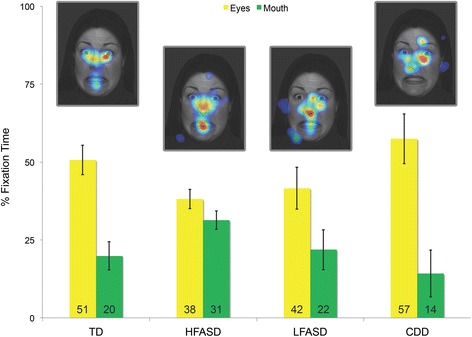
Behavioral analysis through eye tracking. The *yellow* and *green bars* of the graph represent the mean % of time spent fixating (y axis) on the eyes and mouth of the faces, respectively, by cohort (x axis): TD (*n* = 14), HFASD (*n* = 32), LFASD (*n* = 7), CDD (*n* = 5). The *gaze heat maps* illustrate the group-level gaze data overlaid on one of the images at which subjects looked. Compared to TD subjects, HFASD subjects show decreased fixation on the eyes [*t*(44) *=* -2.28, *P* = 0.03, Cohen’s *d* = 0.77] and increased fixation on the mouth [*t*(44) *=* 2.16, *P* = 0.04, Cohen’s *d* = 0.76]. The % of time subjects with LFASD spent looking at the eyes did not differ from HFASD [*t*(37) *=* 0.43, *P =* 0.67, Cohen’s *d* = 0.17], but CDD subjects fixated eyes significantly more than HFASD [*t*(35) = 2.19, *P* = 0.04, Cohen’s *d* = 1.08]. *Error bars* indicate standard error of the mean. All *P* values were calculated by independent *t* test and are two-tailed

We collected eye-tracking data to quantify the social phenotype of our four cohorts [CDD (*n =* 5), LFASD (*n =* 7), HFASD (*n =* 32), and TD (*n =* 14)] as they viewed emotional faces [21]. As shown in Additional file 2: Tables S15 and S16, the groups were not significantly different by sex, age, and total fixation duration on the image. The CDD and LFASD groups were also not significantly different by IQ and autism severity, and the HFASD and TD groups were not significantly different by IQ. As shown in Fig. 6, we replicate prior findings [32–34] of decreased fixation on the eyes [*t*(44) *=* -2.28, *P* = 0.03, Cohen’s *d* = 0.77] and increased fixation on the mouth [*t*(44) *=* 2.16, *P* = 0.04, Cohen’s *d* = 0.76] in HFASD relative to TD. However, while the percentage of time subjects with LFASD spent looking at the eyes did not differ significantly from the HFASD group [*t*(37) *=* 0.43, *P =* 0.67, Cohen’s *d* = 0.17], CDD subjects fixated eyes significantly more than the HFASD group [*t*(35) = 2.19, *P* = 0.04, Cohen’s *d* = 1.08]. Compared to each other, CDD and LFASD subjects did not differ significantly in time spent looking at the eyes [*t*(10) = 1.35, *P* = 0.21, Cohen’s *d* = 0.87]. As with the fMRI results (Fig. 5b), the LFASD group showed an intermediate phenotype to that of the HFASD and CDD groups (eye-mouth ratio, Additional file 2: Table S16).

## Discussion

We are reporting the first multimodal analysis of CDD, a rare form of ASD characterized by late-onset, severe regression. The small cohort size due to its low prevalence, and the challenges of obtaining interpretable data from non-sedated fMRI and eye tracking in subjects with ASD and significant ID necessitated an exploratory study. There is a relative deficiency of reports using these protocols with low-functioning individuals on the autism spectrum. For the first time, a combination of fMRI and eye-tracking analyses are being presented for such individuals to help fill this gap.

Gene expression, neuroimaging, and social behavior analyses suggest that there are neurobiological differences which may underlie the distinct clinical features of CDD. Although no clearly deleterious variants or highly recurrent candidate genes were identified, candidate genes most conserved at variant position or most intolerant of variation, such as *TRRAP*, *ZNF236*, and *KIAA2018*, play a role or may be involved in transcription, which characterizes many ASD-associated genes as well [22]. Gene expression analysis provided some potential insights into CDD. The expression profile of CDD candidate genes resembled that of SSC probands with regression but not SSC probands without regression (matched by IQ), suggesting a pattern relevant to regression. A significant number of CDD candidate genes are co-expressed and may interact in pathways important to the pathophysiology of the disorder. Interestingly, expression of the candidate genes overlapped with face-evoked brain hyperactivity in CDD in non-neocortical regions, such as the thalamus, cerebellum, caudate (striatum), and hippocampus. These regions are known to be involved in distributing eye movements (and thus attention) to socially meaningful stimuli, including faces, early in development. Increased face-evoked activity in CDD was paralleled by increased attention to the eyes of faces, culminating in a normal distribution of attention to the eyes. Still unresolved, though, is how a more typical viewing pattern relates to the poor outcomes which characterize CDD.

Individuals with ASD with greater communicative competence show a more atypical pattern of attention toward faces comprised of decreased looking at the eyes and increased looking at the mouth [35, 36], while individuals with ASD and language impairment have been reported to not differ from typical peers [36]. We found a similar discontinuity in face information processing behaviors, with atypical face-viewing strategies evidenced most clearly for the (most able) HFASD group, and a more typical pattern for the CDD group. While CDD did not differ significantly from LFASD on eye-tracking measures, CDD showed the strongest between group differences in effect sizes referenced against the atypical looking patterns of HFASD. The presence of intact orientation to the eyes and unusual face-sensitive brain activation suggest an alternative developmental pathway for face processing in CDD.

Coinciding with the onset of canonical babbling, the typical infant’s transition from looking at the eyes of a speaker to looking at the mouth is between 4 and 8 months of age [37]. This bias reverts back to the eyes by 12 months for infants viewing people speaking their native language (but not a foreign language), an effect probably driven by advancing expertise and perceptual narrowing. Preferences for looking at mouths in HFASD may reflect higher-order cognitive compensatory mechanisms with scaffolding functions analogous to the 4–8 month transitional period in typical development or biases for second languages later in infancy [33, 38], whereas LFASD and CDD may lie on opposite sides of the 4–8 month divide. The unique face-evoked activity that localized to a set of subcortical structures and the cerebellum in CDD suggests a neoteny in the development of the face-processing system whereby subcortical mechanisms thought to control orienting and attention to faces [39, 40] early in human development remain abnormally involved or cease to be inhibited by top-down regulation following regression. This may represent a marker of the unique developmental process underlying CDD, thereby suggesting a target for studies utilizing eye tracking for early identification and stratification of behaviorally and biologically heterogeneous forms of ASD [36, 41].

It is important to note that our investigations occurred months to years after the onset of symptoms. Since CDD is typically a diagnosis of exclusion, subjects come to our attention for the purposes of research long after the regressive period. Therefore, how the neurobiological features of CDD that we identified relate to the course of regression is unknown. It will be essential to confirm our results in larger cohorts. Ideally, subjects with CDD would be studied before and after the regression to better identify neurobiological correlates; however, this is challenging with such a rare disorder. Since regression is frequently described in ASD, prospective studies of more typical cases of regression may determine whether our results are relevant to regression in the autism spectrum more broadly. It would also be interesting to conduct our studies in regressive disorders such as Rett syndrome. Furthermore, since the fMRI and eye-tracking results revealed that the LFASD subjects had phenotypes intermediate to those of CDD and HFASD, it will be important to study an ID cohort without ASD to better attribute group differences to the effects of ASD versus ID. A major future challenge will be to elucidate the mechanisms by which variants in a set of genes may lead to areas of brain hyperactivity and an apparently normal attention to eyes but, in the end, the severe autism which characterizes CDD.

## Conclusions

In summary, we pursued a multidisciplinary, multi-level approach comprising genetic, brain, and behavioral analyses to conduct an exploratory study of CDD, a rare and severe condition of unclear etiology. Although CDD and other forms of ASD have clinical similarities, the unique natural history of CDD may mark some unique neurobiological features. The clinical and genetic heterogeneity of ASD are well established; our results suggest that there is also heterogeneity of biomarkers, such as affected brain regions and neural circuits. Biomarkers established for high-functioning individuals with ASD may not apply to the substantial proportion of individuals on the spectrum who have ID. Ultimately, the recognition of an increasing number of specific ASD biotypes may translate into more targeted diagnostic tests and treatments.

## Additional files

Additional file 1: Supplementary information includes supplementary methods, references, and figures. (PDF 700 kb)

Additional file 2: Table S1. CDD families for genetic analysis. **Table S2.** Rare non-synonymous and synonymous variants from WES unique to probands or unaffected sibling controls. **Table S3.** Rates of variants from WES in CDD probands and unaffected sibling controls. **Table S4.** Genes represented once in the core probe set (Kang et al. 2011) and used for expression analysis. **Table S5.** Median expression values (Log2-transformed signal intensity) for CDD candidate genes by time period and brain region. **Table S6.** Difference in median expression values (Log2-transformed signal intensity) between non-neocortical and neocortical regions for gene sets by time period. **Table S7.** Pearson correlation coefficients for all pairwise combinations of CDD candidate genes. **Table S8.** Clinical characteristics of subjects studied by neuroimaging. **Table S9.** Features of CDD, LFASD, HFASD, and TD cohorts for neuroimaging analysis. **Table S10.** List of brain regions where TD:discovery exhibits significant faces > houses activation. **Table S11.** Mean % signal change (faces > houses) for each cohort in Fig. 4b. **Table S12.** Mean % signal change (faces > houses) for each cohort in Figure S2. **Table S13.** List of brain regions where CDD exhibits significant faces > houses activation. **Table S14.** Mean % signal change (faces > houses) for each cohort in Fig. 5b. **Table S15.** Clinical characteristics of subjects studied by eye tracking. **Table S16.** Features of CDD, LFASD, HFASD, and TD cohorts in eye-tracking analysis. **Table S17.** Whole-exome sequencing quality metrics. **Table S18.** Features of SSC probands with and without regression by IQ and autism severity. (XLSX 364 kb)

## Abbreviations

AMY: Amygdala
ASD: Autism spectrum disorder
CBC: Cerebellar cortex
CDD: Childhood disintegrative disorder
CNV: Copy number variant
FFG: Fusiform gyrus
fMRI: Functional magnetic resonance imaging
FSIQ: Full-scale IQ
HFASD: High-functioning ASD
HIP: Hippocampus
ID: Intellectual disability
IQ: Intelligence quotient
LFASD: Low-functioning ASD
LGD: Likely gene-disrupting
LOC: Lateral occipital cortex
MD: Mediodorsal nucleus of the thalamus
MFG: Middle frontal gyrus
MTG: Middle temporal gyrus
OMIM: Online Mendelian Inheritance in Man
PG: Precentral gyrus
PhyloP: Phylogenetic *P* value
PolyPhen-2: Polymorphism Phenotyping v2
ROI: Region of interest
SSC: Simons Simplex Collection
STR: Striatum
TD: Typically developing
WES: Whole-exome sequencing
