# Supplementary material for: Neurogenetic analysis of childhood disintegrative disorder
Source: Mol Autism. 2017 Apr 4;8:19. doi: 10.1186/s13229-017-0133-0 (PMC5379515; doi:10.1186/s13229-017-0133-0)
Supplement: Supplementary file 1 — Supplementary information includes supplementary methods, references, and figures. (PDF 700 kb) [file 13229_2017_133_MOESM1_ESM.pdf]

Additional file 1: Supplementary Information

Supplementary methods

Subjects. This research was approved by the Yale University Institutional Review Board, and informed consent was obtained for all subjects. We studied four cohorts: (1) subjects with CDD (n = 17) who were referred to the Yale Child Study Center (YCSC), (2) low-functioning (FSIQ ≤ 75) subjects with ASD (LFASD, n = 12) and early-onset (< 2 years-old) delays, (3) high-functioning (FSIQ ≥ 75) subjects with ASD (HFASD, n = 50) and early-onset (< 2 years-old) delays, and (4) typically-developing subjects (TD, n = 26). A multidisciplinary (child psychiatrist, developmental-behavioral pediatrician, child psychologist, and speech/language pathologist) team of expert clinicians at the YCSC evaluated records for each child with suspected ASD to determine whether they met DSM-IV criteria for ASD (to remain consistent with the use of the DSM-IV to diagnose CDD). Clinical judgment was supplemented with the Autism Diagnostic Observation Schedule (ADOS) and the Autism Diagnostic Interview-Revised (ADI-R). Given the challenge of differentiating ASD and ID from ID alone in very low-functioning individuals and the limitations of current diagnostic instruments for this population, the differential diagnosis was made based on the consensus clinical judgment of experienced clinicians specializing in low-functioning individuals with ASD and ID and with ID alone. In addition, for suspected CDD, expert clinicians (child psychiatrist, developmental-behavioral pediatrician, child neurologist, and/or child psychologist) conducted a comprehensive interview with parents and viewed any available home videos to characterize the nature of each subject's possible regression. Besides meeting full criteria for ASD, CDD was defined as: (1) Loss of language skills after previous period of typical development. The child must have used at least two-word phrases daily and spontaneously prior to the regression. We required language loss, as it is the most objective and quantifiable domain as reported by parents. (2) Loss in at least one other domain: social skills or adaptive behavior, bowel or bladder control, play, and motor skills (reflective of the DSM-IV criteria for CDD). (3) Loss must have occurred after 24 months of age. (4) The child must not have regained level of skill prior to loss. (5) FSIQ ≤ 75. Although loss of cognitive ability was not part of the DSM-IV criteria for CDD, severe regression is most noticeable in the cognitive domain, and, in practice, we only diagnose CDD when there is comorbid ID.

DNA samples. Genomic DNA was prepared from 15 families affected by CDD (Additional file 2: Table S1). Both biological parents were available for all probands as well as 13 unaffected siblings. DNA was extracted from blood for all families except CDD07, for which only lymphoblastoid cell line DNA was available. For CDD21 and CDD22, only whole-genome amplified DNA from blood was available.

Whole-exome sequencing and quality control. All DNA samples were sequenced at the Yale Center for Genome Analysis. Exonic sequences were selected by the NimbleGen v2.0 exome capture reagent (Roche, Basel, Switzerland) and sequenced on the HiSeq 2000 (75 bp paired-end reads; Illumina, San Diego, CA, USA). Reads were mapped to the human reference genome (hg19) using CASAVA v1.8 (ELAND v2). Quality metrics are shown in Additional file 2: Table S17, indicating high quality whole-exome sequencing data. Single nucleotide variants and small insertions/deletions (indels) were identified and assigned quality scores (QS) using SAMtools (http://samtools.sourceforge.net/). All variants were annotated for impact on the encoded protein (synonymous, missense, nonsense, splice site, frameshift) and frequency using dbSNP141/1000 Genomes (May 2011 release), NHLBI GO ESP Exome Variant Server (ESP6500SI-V2), and an in-house database of 2500 exomes. Each exome matched the recorded sex of the subject. Family relationships were validated using an in-house Perl script comparing the overlap of novel heterozygous variants between members of each family. All reported family structures were confirmed.

Ancestry mapping. EIGENSTRAT (http://genepath.med.harvard.edu/~reich/EIGENSTRAT.htm) was used to compare SNP genotypes of CDD family members to individuals of known ancestry in HapMap3 (http://hapmap.ncbi.nlm.nih.gov/). 1,824 SNPs from whole-exome sequencing data were pre-defined: (1) minor allele frequency (MAF) > 5%, (2) not in significant linkage disequilibrium with other SNPs analyzed,

(3) 100 kb apart, (4) not in a region of segmental duplication, (5) satisfy Hardy-Weinberg equilibrium ($P < 0.001$), and (6) contain high Fst values (different frequencies across major ethnic groups in HapMap3). Eigenvalues of the first two principal components, which contributed the greatest amount of variation relative to the other principal components, were plotted against each other (Additional file 1: Fig. S3). The principal component analysis correctly separated and distinguished ancestry groups in HapMap3 samples and confirmed the self-reported race and ethnicities of the subjects (Additional file 2: Table S1).

Genotyping. Subjects were genotyped using the HumanOmni2.5M BeadChip (Illumina). All DNA samples were hybridized and scanned simultaneously on the Illumina iScan to minimize batch effects and variation. All subjects had a genotyping call rate > 95%. Genotyping data were analyzed by PLINK v1.07 [1] (http://pngu.mgh.harvard.edu/~purcell/plink/) and confirmed the recorded sex and family relationships of each subject.

***De novo* and inherited sequence variant detection.** Three types of rare [novel or found at most once across 1000 Genomes (May 2011 release), NHLBI GO ESP Exome Variant Server (ESP6500SI-V2), and in-house database of 2500 exomes] protein-changing variants from whole-exome sequencing were prioritized for study: (1) *de novo*, (2) homozygous, and (3) hemizygous (mother-to-son transmission on chrX). The Exome Aggregation Consortium (ExAC) Browser was not used to filter out variants by frequency since approximately 23% of subjects come from neuropsychiatric studies (exac.broadinstitute.org/faq); still, all of our genotypes of interest (*de novo*, homozygous, or hemizygous) have frequency $\leq$ 0.36% in this database. All *de novo* variants were confirmed by Sanger sequencing in both forward and reverse directions. All homozygous and hemizygous variants at $\leq$ 1% general population frequency that were unique to probands (not shared by unaffected siblings) were visualized by in-silico inspection and/or Sanger validated. Sequencing chromatograms were aligned and analyzed using Sequencher v4.9 (Gene Codes, Ann Arbor, MI, USA).

De novo variants were identified using a Bayesian algorithm as previously described [2]. Virtually 100% of *de novo* variants with a Bayesian quality score (BQS) $\geq$ 50 validate by Sanger sequencing [2]. For the purposes of comparing *de novo* rates between probands and siblings, only variants with a BQS $\geq$ 50 were considered. To maximize our discovery of *de novo* variants in probands, all variants with a BQS $\geq$ 1 were both inspected computationally by the visualization of plot reads and by Sanger sequencing. 100% (5/5) of *de novo* variants with a BQS $\geq$ 50 and 11% (2/18) with a BQS between 1 and 50 were confirmed.

Homozygous variants were required to have: (1) SAMtools QS $\geq$ 60 (94% of such variants confirm by Sanger sequencing in our experience), (2) heterozygous genotypes (SAMtools QS $\geq$ 60) in both parents, and (3) the homozygous genotype seen at most once in 1000 Genomes (May 2011 release) and NHLBI GO ESP Exome Variant Server (ESP6500SI-V2).

In male probands, hemizygous variants (mother-to-son transmission on chrX) were required to have: (1) SAMtools QS $\geq$ 100, (2) the proband's father was required to have the hemizygous reference genotype and the proband's mother was required to have a heterozygous genotype with SAMtools QS $\geq$ 100, and (3) the hemizygous (in males) and homozygous (in females) genotypes seen at most once in 1000 Genomes (May 2011 release) and NHLBI GO ESP Exome Variant Server (ESP6500SI-V2).

Copy number variant (CNV) detection. Three types of rare (novel or seen at most once in the Database of Genomic Variants) CNVs from genotyping data were prioritized for study: (1) *de novo*, (2) homozygous, and (3) hemizgyous (mother-to-son transmission on chrX). CNV detection was performed using three algorithms, PennCNV Revision 220, QuantiSNP v1.1, and GNOSIS, as previously described [3]. PennCNV and QuantiSNP are based on the Hidden Markov Model. GNOSIS uses a continuous distribution function to fit the intensity values from the HapMap data and determine thresholds for significant points in the tails of the distribution that are used to detect copy number changes. Analysis and merging of the CNV predictions were performed using an in-house Perl script. All rare ($\geq$ 50% of CNV at $\leq$ 1% frequency in the Database of Genomic Variants) genic CNVs that were unique to probands (not shared by unaffected siblings) and predicted by at least PennCNV and QuantiSNP were tested by quantitative PCR (qPCR) as

previously described [3].

Gene expression levels. Gene-level brain expression data (Platform GPL5175, Affymetrix GeneChip Human Exon 1.0 ST Array) [4], which were generated as part of the BrainSpan project (www.hbatlas.org), were downloaded from the NCBI GEO database (accession number GSE25219) in the form of log_2-transformed signal intensity values. Affymetrix uses background probes with matching GC content for background correction for all probes on the array (http://media.affymetrix.com/support/technical/white papers/exon_background_correction_whitepaper.pdf). Genes represented once in the core probe set were identified for the following groups: (1) all nonsynonymous variants (*n* = 40) in CDD probands (*n* = 15), (2) all synonymous variants (*n* = 16) in CDD probands (*n* = 15), (3) all nonsynonymous variants (*n* = 17) in unaffected siblings (*n* = 13) of CDD probands, (4) all synonymous variants (*n* = 8) in unaffected siblings (*n* = 13) of CDD probands, (5) *de novo* nonsynonymous variants (*n* = 123) in SSC probands with regression (*n* = 257) [5], (6) *de novo* synonymous variants (*n* = 37) in SSC probands with regression (*n* = 257) [5], (7) *de novo* nonsynonymous variants (*n* = 132) in SSC probands without regression (*n* = 249) [5], (8) *de novo* synonymous variants (*n* = 52) in SSC probands without regression (*n* = 249) [5], (9) *de novo* nonsynonymous variants (*n* = 1526) in SSC probands (*n* = 2508) [5], (10) *de novo* synonymous variants (*n* = 503) in SSC probands (*n* = 2508) [5], (11) *de novo* nonsynonymous variants (*n* = 1044) in unaffected siblings (*n* = 1911) of SSC probands [5], (12) *de novo* synonymous variants (*n* = 389) in unaffected siblings (*n* = 1911) of SSC probands [5], (13) *de novo* LGD variants (*n* = 297) in SSC probands (*n* = 2508) [5], (14) *de novo* LGD variants (*n* = 156) in unaffected siblings (*n* = 1911) of SSC probands [5], (15) highest-risk genes (*n* = 67) in SSC, ASC, and AGP probands (*n* = 8009) [5-7], and (16) all genes in the BrainSpan dataset (*n* = 16947) [4] (Additional file 2: Table S4). Since the published ASD candidate genes identified by WES and CNV studies [5-7] were not filtered by positive brain expression as determined by BrainSpan, we did not filter by this parameter either across the 16 groups to maintain consistency. To identify SSC probands with and without regression, we queried the SSC v.14 Phenotype Data Set (https://sfari.org/resources/sfari-base). SSC probands with regression were defined as individuals who received maximal scores on two questions from the ADI-R: (1) Question #11 loss of language skills after acquisition: Were you ever concerned that [subject] might have lost language skills during the first years of her/his life? Was there ever a time that s/he stopped speaking for some months after having learned to talk? (0=No, 1=Yes) and (2) Question #20 loss of skills (for at least 3 months): Has there ever been a period when [subject] seemed to get markedly worse or dropped further behind in her/his development? (0=no consistent loss of skills, 1=probable loss of skill but of a degree that falls short of specified criteria, 2=account of definite loss of skills over a period of time). Therefore, SSC probands with regression were defined as individuals with a total score of 3. SSC probands without regression were defined as individuals who received scores of 0 on both questions. SSC probands with and without regression were matched by sex, age at evaluation, IQ, and autism symptom severity (Additional file 2: Table S18). Equality of variances was determined by Levene's test.

The median expression value for genes affected by nonsynonymous variants in CDD probands and represented once in the core probe set (*n* = 40) across all brain samples was determined and plotted using ggplot2 in R for each brain region (NCX, HIP, AMY, STR, MD, CBC) and time period. The difference in median expression values between non-neocortical (HIP, AMY, STR, MD, CBC) and neocortical (NCX) brain regions for all 16 gene groups described above was also plotted using ggplot in R for each time period. Local polynomial regression fitting was used to smooth the scatter plots. This difference reached a maximum value at period 6 for genes affected by nonsynonymous variants in CDD probands (Fig. 2). Permutation testing with 100,000 iterations was performed to determine the significance of this difference. Random sets of 40 genes were selected from the BrainSpan dataset, and the differential expression between non-neocortical and neocortical brain regions was calculated at period 6. The *P* value was determined by the number of times the differential expression was greater than or equal to the difference observed for CDD candidate genes.

Gene coexpression analysis. For the 40 CDD candidate genes, a gene coexpression matrix was constructed

using the mean expression value for each gene in each brain region for each brain sample and calculating the Pearson correlation coefficient for each pairwise gene combination across all data points. Two genes were considered coexpressed if they had a correlation coefficient $r \geq 0.7$. The number of genes that were coexpressed with at least one other gene from the set as well as the number of correlations/gene and the mean coefficient value were determined. Permutation testing with 100,000 iterations of 40 random genes from the BrainSpan dataset was performed to determine the significance of these values. The *P* value was calculated by the number of iterations that resulted in a greater or equal number of genes being coexpressed, correlations/gene, and mean coefficient value. To visualize the gene coexpression network, edges were drawn between two genes if their correlation coefficient $r \geq 0.7$, using the organic layout function of Cytoscape [8]. Positive correlations are shown in blue, and negative correlations are shown in red. The greater the magnitude of the coefficient, the wider and darker are the edges. The size of a node is proportional to the number of edges the node has.

Non-sedated fMRI data acquisition and paradigm. Images were collected on a Siemens 3T Tim Trio scanner located at the Yale University Magnetic Resonance Research Center. High-resolution, T1-weighted anatomical images were acquired using a magnetization-prepared rapid gradient echo (MPRAGE) sequence (TR = 1,900 ms, TE = 2.96 ms, flip angle = 9°, matrix = 256×256, voxel size = 1x1 mm^2, field of view = 256×256 mm^2, slice thickness = 1.00 mm, 160 slices, interleaved acquisition). Whole-brain functional images were acquired using a single-shot, gradient-recalled echo planar pulse sequence (TR = 2,000 ms, TE = 25 ms, flip angle = 60°, matrix = 64×64, voxel size = 3.44×3.44 mm^2, field of view = 220×220 mm^2, slice thickness = 4.00 mm, 34 slices, interleaved acquisition) sensitive to blood oxygenation level-dependent (BOLD) contrast. The fMRI task consisted of ten 12 s blocks of images (5 blocks of fearful faces and 5 blocks of houses). The blocks, consisting of either faces or houses, alternated, with the fearful faces presented first. Each block included six images and each image was presented for 2 s.

fMRI data collection, processing, and analyses. The neurobiology of CDD has not been elucidated in part due to the technical difficulty of conducting experimental protocols with very low-functioning subjects. To obtain this data, we implemented an individualized training protocol to accustom subjects to the scanner environment as well as to provide training and reinforcement for compliance with the requirement to remain very still during fMRI and eye tracking. We utilized the following training procedures: (1) preparation for scanning and eye tracking through videos sent home before the visits; (2) preparation for use of earphones and earplugs in the scanner by sending home earphones and earplugs and asking parents to help their children learn to wear them properly for increasing periods of time; (3) providing a list of “statue” and “let’s-take-a-picture” games for parents to engage their children in at home before and between the training protocol sessions, to help children learn to “pretend to be a statue”/“pretend to have a picture taken,” involving earning rewards for holding still for increasing lengths of time; (4) gradual introduction to experimental procedures through interaction with, first, a “toy” scanner used on a stuffed animal, then a mock scanner before entering the scanning environment; (5) helping subjects become familiar and comfortable with the pictures to be presented in the scanner by providing analogues of all stimuli, first on a tabletop, then in a mock scanner before moving to the scanner; (6) using picture schedules to accompany mock scanner sessions and as reminders prior to scanning and eye-tracking sessions; (7) utilizing visual transition signals between “statue”/”picture taking” and “move” conditions; (8) providing comforting activities and rewards to assist children in overcoming distress, along with parental support. Approximately 70% of low-functioning subjects who were able to progress to the real scanner were able to give usable data. Across all cohorts, no subject had an active seizure disorder, since this is an exclusion criterion for our MRI studies. Subjects were not taking medications that are known to affect the fMRI BOLD signal.

Data were processed and analyzed using FEAT v6.00 (FMRI Feat Analysis Tool) of FSL 5.0.6, via a data processing pipeline implemented in the Yale University High-Performance Computing clusters. The pipeline consisted of: (1) motion correction using MCFLIRT, (2) interleaved slice timing correction, (3) BET brain extraction, (4) spatial smoothing using a kernel of FWHM 5 mm, and (5) high-pass temporal filtering using 100 s. The first and last 10 volumes were fixation (no images were presented). The remaining

60 volumes were analyzed.

The EPI data was registered to the subject's structural scan (with the brain extracted using BET) via linear boundary-based registration and then registered to the MNI152 standard brain with linear transformation with 12 degrees of freedom. Artifact removal was performed with FSL's FIX (FMRIB's ICA-based Xnoiseifier). The standard denoising classifier from FSL's FIX package was applied to the raw results from FSL's MELODIC ICA (Independent Component Analysis) to identify artifact components such as head movement, respiratory motion, and scanner artifacts. General Linear Model (GLM)-based analyses, where normality is assumed, were conducted for each subject to assess task-related BOLD responses. We did not include motion regressors in GLM to avoid over (duplicated) correction of head movement. To create predictors for fearful faces and houses conditions, the timing of the corresponding blocks (onset in seconds, duration = 12 s, weighting = 1) was convolved with the default gamma function (phase = 0 s, standard deviation = 3 s, mean lag = 6 s) with temporal derivatives. Time series autocorrelation was estimated using FSL's FILM pre-whitening. Due to the limited number of low-functioning subjects with usable fMRI data, we conservatively limited the statistical inference to our data only and used fixed-effects analysis in the fMRI group analysis [9]. The subject-level parameter estimates were inputs for the group-level fMRI analyses. Correction for multiple comparisons was implemented with a highly stringent voxel-level threshold of $Z > 3.09$ ($P < 0.001$, one-sided) and a cluster-level threshold of $P < 0.05$ for the main whole-brain analysis, or $Z > 2.58$ ($P < 0.01$, two-sided) for the analysis within FFG and a cluster-level threshold of $P < 0.05$.

Eye-tracking. Eye-tracking data were collected using a Tobii T60 XL monitor-integrated eye tracker. Subjects sat in front of a computer monitor and viewed static images of emotional faces. The images were photographs of 14 adult male and female actors centered on a neutral backdrop (extracted from the NimStim Face Stimulus set) [10] making three different expressions: happy, fearful, or neutral. All stimuli were grayscale, with the mean luminosity of each image adjusted to equal 80% of maximal brightness using Adobe Photoshop. Images were 506 pixels (11.4 degrees) wide and 649 pixels (14.6 degrees) high. Subjects first saw a white fixation cross, centered in the screen for 4 seconds, followed by images from the stimulus set lasting 2 seconds, alternating with a screen with a fixation cross only, lasting either 2 or 3 seconds. The location of the fixation cross varied, appearing at any of the corners of the screen. The purpose of the variations in location and duration of the fixation cross was to encourage subjects to alter the part of the screen they were looking at between stimuli images, so that when they were faced with a new stimulus they would have to reorient their eyes. Faces appeared for a total of 84 s during the paradigm. The experiment was administered two times over consecutive sessions for each subject. Regions of interest for the eyes and mouth were manually defined on the face images and were equal in size across all images of faces.

Approximately 80% of low-functioning subjects who were able to sit in front of the monitor gave usable data. Valid trials were defined as those for which data retention was $\geq$ 50%, with data retention calculated by dividing the number of eye-tracking samples that were identified by Tobii Studio as valid by the total number of samples over which the stimulus was presented. The variables of primary interest were total fixation duration on the image (TFD) as well as the amount of time spent looking at eyes and mouth, separately. In order to adjust for the probability that not all subjects would look at the face images for an equal amount of time, analyses of eye and mouth time were based on the ratio of time spent looking at eyes to the overall face (%Eye), and the ratio of time spent looking at mouth to the overall face (%Mouth). Statistical analyses involved an analysis of variance (ANOVA) approach, with independent sample t-tests for subsequent planned comparisons. Equality of variances was tested using Levene's Test, with subsequent Welch-Satterthwaite correction for degrees of freedom as appropriate. Calibration and calibration quality checks were conducted using Tobii's in-software verification tools (Tobii Studio) with a standard 5-point calibration.

Gaze heat maps were constructed with MATLAB scripts that provided visualization of group-level gaze data overlaid on the images presented to subjects. For each presented stimulus, the associated gaze-points upon that stimulus were collected from all subjects in a given group and spatial smoothed using a Gaussian filter with an approximately 2×2 degree kernel window, which had a standard deviation of

approximately 0.5 degrees.

Statistical methods. *Sample size.* No statistical methods were used to predetermine sample sizes. CDD, LFASD, HFASD, and TD cohort sizes represent the maximum number of subjects who could be recruited and give successful fMRI and/or eye-tracking data. SSC probands with and without regression cohort sizes represent the maximum number of subjects who met ADI-R criteria for regression or no regression, for whom WES data were available, and who could be matched by sex, age at study, IQ, and autism symptom severity.

Genetics. Statistical analysis was performed in R (version 3.2.0). Mutation burden analysis between CDD probands and their unaffected siblings was performed using Fisher exact test (Additional file 2: Table S3). Permutation testing with 100,000 iterations was performed to determine the significance of differential gene expression between non-neocortical and neocortical brain regions for CDD candidate genes (Fig. 2) and for the co-expression analysis (Fig. 3). Equality of variances for SSC probands with and without regression was determined by Levene's test. They were matched by sex, age at study, IQ, and autism symptom severity, as determined by the Fisher exact test or independent t-test as appropriate (Additional file 2: Table S18). All P values are two-tailed.

Neuroimaging. Cohorts were compared by sex, age at study, IQ, autism symptom severity, intracranial volume, and relative head motion in the scanner, as determined by chi-square, one-way ANOVA, or independent t-test as appropriate (Additional file 2: Table S9). fMRI analyses involved the commonly employed standard parametric GLM approach in FSL (version 5.0.6), where normality is assumed. Due to the limited number of low-functioning subjects with usable fMRI data, we conservatively limited the statistical inference to our data only and used fixed-effects analysis in the fMRI group analysis [9]. The subject-level parameter estimates were inputs for the group-level fMRI analyses. Correction for multiple comparisons was implemented with a highly stringent voxel-level threshold of $Z > 3.09$ ($P < 0.001$, one-sided) and a cluster-level threshold of $P < 0.05$ for the main whole-brain analysis, or $Z > 2.58$ ($P < 0.01$, two-sided) for the analysis within FFG and a cluster-level threshold of $P < 0.05$. The independent t-test was used for subsequent planned comparisons, and P values are two-tailed. All bar graphs show mean and standard error of the mean.

Eye-tracking. Cohorts were compared by sex, age at study, IQ, autism symptom severity, and total fixation duration on the image, as determined by chi-square, one-way ANOVA, or independent t-test as appropriate (Additional file 2: Table S16). Statistical analyses involved an analysis of variance (ANOVA) approach, with independent sample t-tests for subsequent planned comparisons (Additional file 2: Table S16). Equality of variances were tested using Levene's Test, with subsequent Welch-Satterthwaite correction for degrees of freedom as appropriate. All P values are two-tailed. All bar graphs show mean and standard error of the mean.

Supplementary references

1. Purcell S, Neale B, Todd-Brown K, Thomas L, Ferreira MA, Bender D, et al. PLINK: a tool set for whole-genome association and population-based linkage analyses. Am J Hum Genet. 2007;81:559-75.
2. Zaidi S, Choi M, Wakimoto H, Ma L, Jiang J, Overton JD, et al. De novo mutations in histone-modifying genes in congenital heart disease. Nature. 2013;498:220-3.
3. Sanders SJ, Ercan-Sencicek AG, Hus V, Luo R, Murtha MT, Moreno-De-Luca D, et al. Multiple recurrent de novo CNVs, including duplications of the 7q11.23 Williams syndrome region, are strongly associated with autism. Neuron. 2011;70:863-85.
4. Kang HJ, Kawasawa YI, Cheng F, Zhu Y, Xu X, Li M, et al. Spatio-temporal transcriptome of the human brain. Nature. 2011;478:483-9.
5. Iossifov I, O'Roak BJ, Sanders SJ, Ronemus M, Krumm N, Levy D, et al. The contribution of de novo coding mutations to autism spectrum disorder. Nature. 2014;515:216-21.
6. De Rubeis S, He X, Goldberg AP, Poultney CS, Samocha K, Cicek AE, et al. Synaptic, transcriptional and chromatin genes disrupted in autism. Nature. 2014;515:209-15.

7. Sanders SJ, He X, Willsey AJ, Ercan-Sencicek AG, Samocha KE, Cicek AE, et al. Insights into autism spectrum disorder genomic architecture and biology from 71 risk loci. Neuron. 2015;87:1215-33.
8. Cline MS, Smoot M, Cerami E, Kuchinsky A, Landys N, Workman C, et al. Integration of biological networks and gene expression data using Cytoscape. Nature Protoc. 2007;2:2366-82.
9. Desmond JE, Glover GH. Estimating sample size in functional MRI (fMRI) neuroimaging studies: statistical power analyses. J Neurosci Methods. 2002;118:115-28.
10. Tottenham N, Tanaka JW, Leon AC, McCarry T, Nurse M, Hare TA, et al. The NimStim set of facial expressions: judgments from untrained research participants. Psychiatry Res. 2009;168:242-9.

Supplementary figures

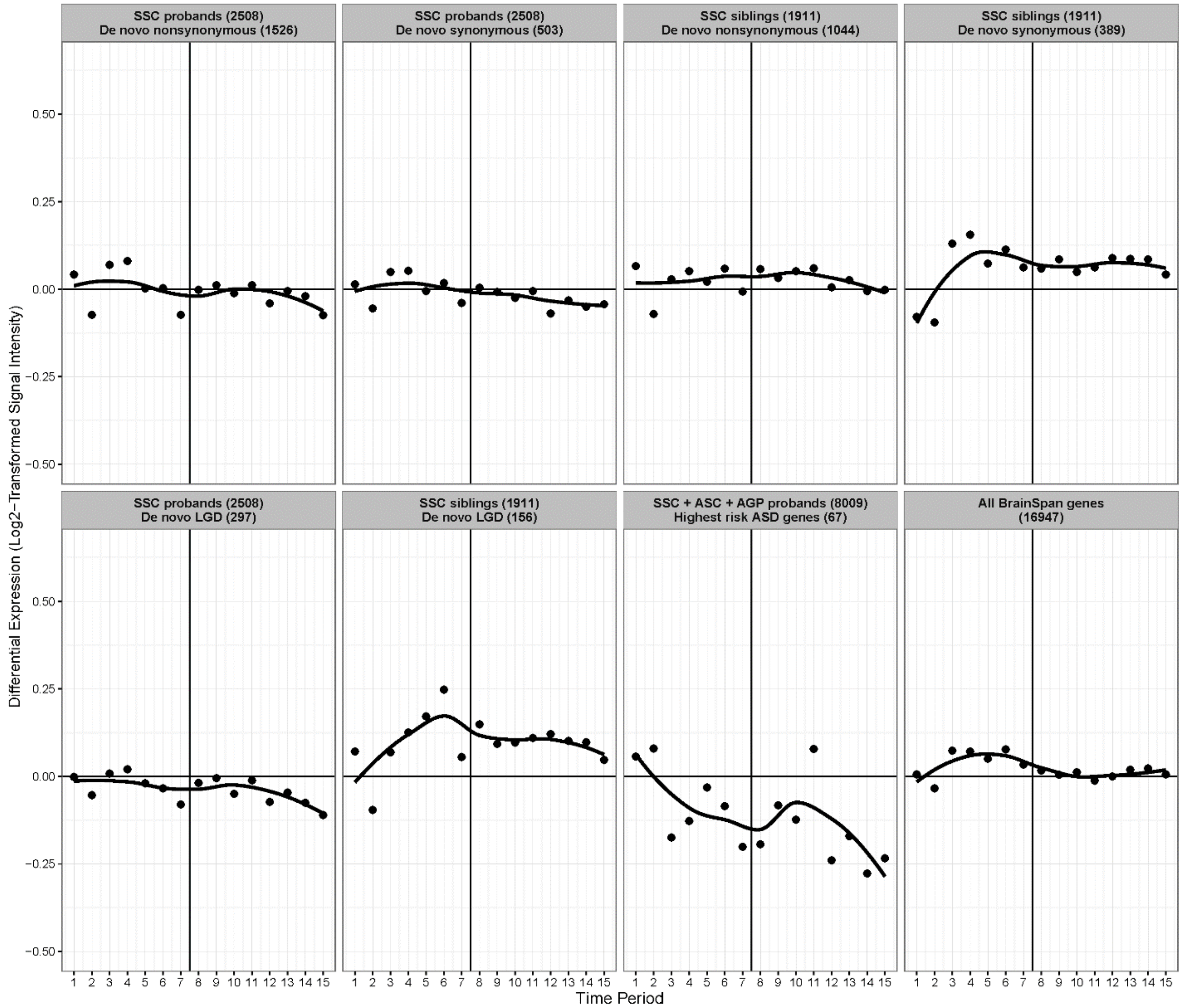

Fig. S1 Differential expression levels of various gene sets. The difference in expression levels (non-neocortical minus neocortical brain regions) is shown for genes affected by nonsynonymous, synonymous, and LGD variants in SSC probands and their unaffected siblings. Also plotted are data for genes most significantly associated with ASD by three recent, large WES and CNV studies [5-7] and all genes in the BrainSpan dataset [4]. The dark vertical line in each panel indicates birth. The number in parentheses indicates the number of subjects or variants. AGP, Autism Genome Project; ASC, Autism Sequencing Consortium; LGD, likely gene disrupting; SSC, Simons Simplex Collection.

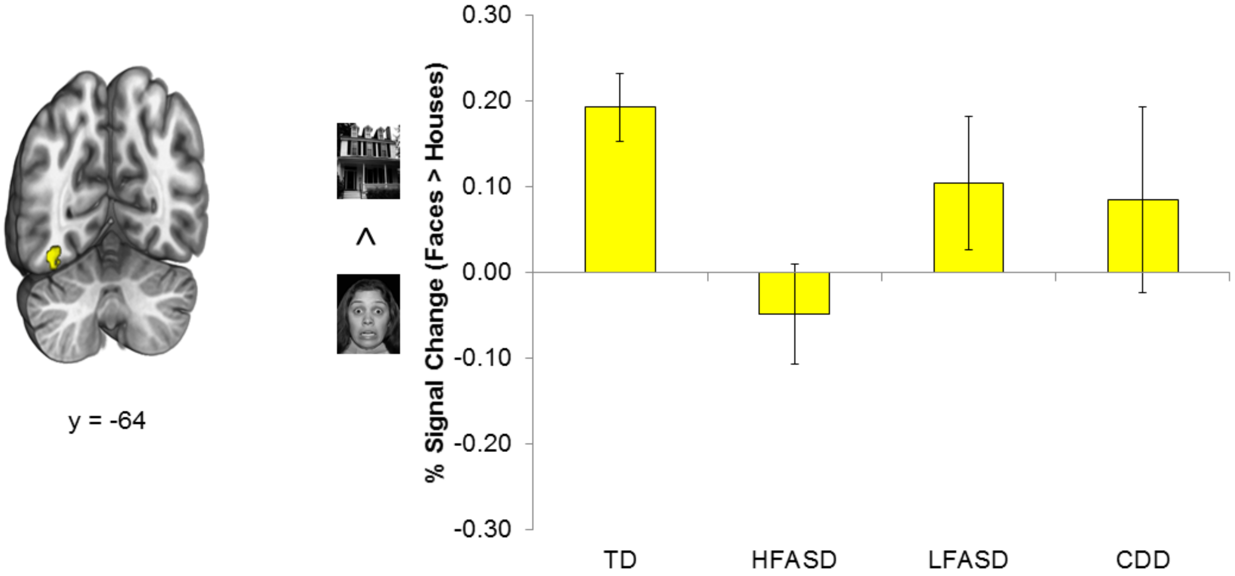

Fig. S2 Comparison of fMRI faces > houses activity in a region within the middle fusiform gyrus (FFG) corresponding to the expected location of the fusiform face area. This region is defined by TD > HFASD in FFG. Left: The yellow color brain map indicates TD > HFASD activity within the FFG (an example slice taken at MNI152 y = -64 mm, 50 voxels), $Z > 2.58$, whole-brain corrected at the cluster-level $P < 0.05$. Right: mean % signal change from the faces > houses contrast within the region of TD > HFASD in the FFG [$t(31) = 3.54$, $P = 0.0013$, Cohen's $d = 1.29$] by all groups: TD ($n = 19$), HFASD ($n = 14$), LFASD ($n = 7$), and CDD ($n = 7$). Comparison of CDD relative to TD revealed no significant difference [$t(24) = 1.18$, $P = 0.25$, Cohen's $d = 0.54$], as did LFASD relative to TD [$t(24) = 1.10$, $P = 0.28$, Cohen's $d = 0.51$]. Error bars indicate standard error of the mean. All P values were calculated by independent t-test and are two-tailed.

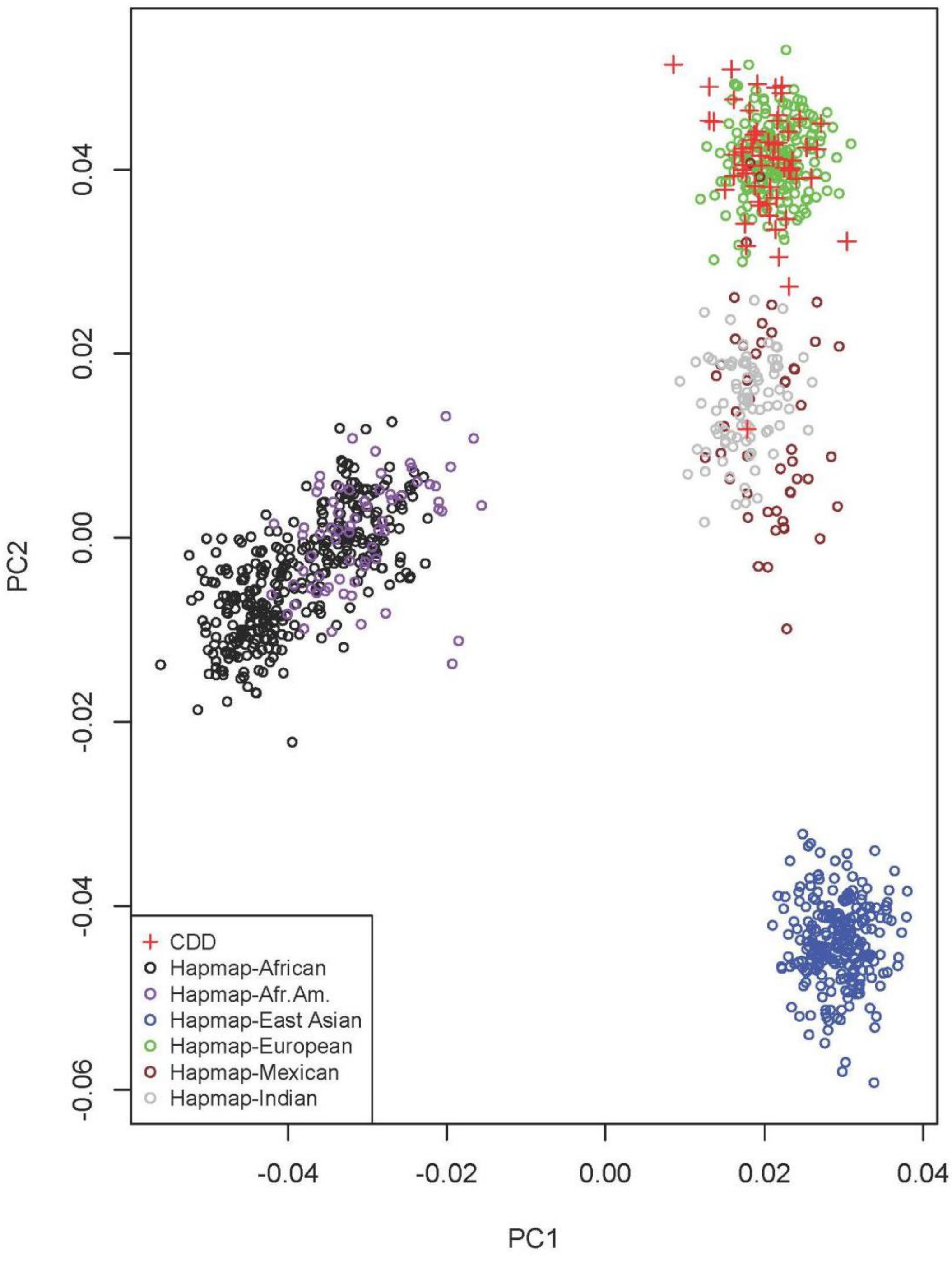

Fig. S3 EIGENSTRAT was used to compare SNP genotypes of CDD family members to individuals of known ancestry in HapMap3. Eigenvalues of the first two principal components (PC1 and PC2), which contributed the greatest amount of variation relative to the other principal components, were plotted against each other.
